# The NADPH oxidase NOX4 represses epithelial to amoeboid transition and efficient tumour dissemination

**DOI:** 10.1038/onc.2016.454

**Published:** 2016-12-12

**Authors:** E Crosas-Molist, E Bertran, I Rodriguez-Hernandez, C Herraiz, G Cantelli, À Fabra, V Sanz-Moreno, I Fabregat

**Affiliations:** 1 Molecular Oncology, Bellvitge Biomedical Research Institute (IDIBELL), L'Hospitalet de Llobregat, Barcelona, Spain; 2 Tumour Plasticity Laboratory, Randall Division of Cell and Molecular Biophysics, New Hunt's House, Guy's Campus, King's College London, London, UK; 3 Departament de Ciències Fisiològiques II, University of Barcelona, Barcelona, Spain

## Abstract

Epithelial to mesenchymal transition is a common event during tumour dissemination. However, direct epithelial to amoeboid transition has not been characterized to date. Here we provide evidence that cells from hepatocellular carcinoma (HCC), a highly metastatic cancer, undergo epithelial to amoeboid transition in physiological environments, such as organoids or three-dimensional complex matrices. Furthermore, the NADPH oxidase NOX4 inhibits this transition and therefore suppresses efficient amoeboid bleb-based invasion. Moreover, NOX4 expression is associated with E-cadherin levels and inversely correlated with invasive features. NOX4 is necessary to maintain parenchymal structures, increase cell–cell and cell-to-matrix adhesion, and impair actomyosin contractility and amoeboid invasion. Importantly, NOX4 gene deletions are frequent in HCC patients, correlating with higher tumour grade. Contrary to that observed in mesenchymal cell types, here NOX4 suppresses Rho and Cdc42 GTPase expression and downstream actomyosin contractility. In HCC patients, *NOX4* expression inversely correlates with *RhoC* and *Cdc42* levels. Moreover, low expression of *NOX4* combined with high expression of either *RhoC* or *Cdc42* is associated with worse prognosis. Therefore, loss of NOX4 increases actomyosin levels and favours an epithelial to amoeboid transition contributing to tumour aggressiveness.

## Introduction

Metastatic dissemination is the main cause of cancer deaths. Cell migration and invasion underlie the complex set of events that are required for metastasis to succeed. Cancer cells can disseminate from the primary tumour either as individual cells, using amoeboid or mesenchymal type of movement, or as cell sheets, strands and clusters using collective migration.^1^ Individual cell migration appears to be required for blood borne metastasis.^2^ Different types of individual movement differ in their cell–matrix adhesion requirements, a process that is regulated by integrins and their engagement of Rho GTPase signalling. Rho GTPases are key regulators of cell migration due to their actions on the cytoskeleton. High levels of actomyosin contractility and lower levels of adhesion are characteristic of rounded amoeboid form of movement, in which blebs are used as functional protrusions.^3, 4^ Actomyosin contractility in amoeboid migration can be regulated either by Rho and downstream ROCK activity, or by Cdc42 through PAKs,^5, 6, 7, 8^ in both cases resulting in phosphorylation of MLC2 and therefore activating myosin II.^9^ In contrast, elongated mesenchymal migrating cells use Rac-dependent actin polymerisation, and higher levels of integrin-dependent adhesion.^10, 11, 12^ Intravital *in vivo* imaging studies have revealed how amoeboid migration is the fastest way of moving, being the preferred strategy used in the invasive fronts of melanomas and breast cancers.^11, 12, 13, 14^ This is due to the lower adhesive requirements that allow actin cortex flows.^15^ Furthermore, physical confinement imposed by physiologically relevant complex matrices favours amoeboid behaviour.^14, 16, 17^ Therefore, understanding if amoeboid strategies can be used by other cancer types is crucial.

Hepatocellular carcinoma (HCC) is the most frequent liver tumour, presenting a high frequency of relapse and metastasis.^18, 19^ Molecular markers are not used in diagnosis or determination of prognosis and treatment for patients; indeed, studies now aim to identify molecular mechanisms that allow the design of new biomarkers at earlier stages and better predict their survival time and the adequacy of treatment.^19^ Studies on HCC cell migration have been mainly focused on the role of epithelial–mesenchymal transition (EMT) and its relevance in the metastatic process.^20^ During EMT an epithelial cell loses cell–cell junctions and acquires a mesenchymal-like phenotype, which increases its migratory and invasive properties. This phenomenon takes place particularly during embryogenesis and cancer^21^ and is regulated by numerous signalling pathways,^22^ which finally converge in the expression of transcription factors that regulate EMT.^23^ Cancer cells undergoing EMT have lost E-cadherin junctions and may move as individual cells. However, there is a lack of knowledge regarding the types of movement that contribute to HCC metastatic competence.

During cell migration, Rho GTPases, reactive oxygen species (ROS) and cytoskeletal organisation appear to function as a complex regulatory network; however, more work is needed to fully elucidate the interactions between these factors and their potential *in vivo* relevance.^24^ The NADPH oxidase (NOX) family has emerged in the last years as an important source of ROS in signal transduction.^25, 26^ In the liver, NOX4 plays important roles mediating transforming growth factor-beta (TGF-β) actions. In stellate cells, NOX4 is required for TGF-β-induced myofibroblast activation, contributing to the development of liver fibrosis,^26^ which attracted interest in the development of NOX inhibitors that could be used in the clinic to ameliorate this disease.^27^ However, in hepatocytes and liver tumour cells, NOX4 mediates TGF-β-induced mitochondrial-mediated apoptosis, through modulation of the expression of the pro-apoptotic genes BIM and BMF,^28^ which contributes to its well-known tumour suppressor effects. Therefore, inhibition of NOX4 in liver cells might lead to pro-tumorigenic processes. In favour of this hypothesis, we recently found that NOX4 plays a role in regulating liver cell proliferation either under physiological conditions or during tumorigenesis.^29^ NOX4 silencing increases the tumorigenic potential of human HCC cells in xenografts in mice, resulting in earlier onset of tumour formation and increase in tumour size.^29^ Overall, results strongly suggested that NOX4 plays a growth inhibitory role. However, whether NOX4 could also regulate other cellular processes that occur later in progression and that favour tumour metastasis, such as migration and invasion, remains unknown.

In the current study, we have explored the role of NOX4 in regulating HCC migration and invasion. We have found that NOX4 supports epithelial parenchymal structures and that NOX4 loss, a common event in HCC, is accompanied by acquision of efficient amoeboid invasive behaviour.

## Results

### NOX4 is important for maintaining epithelial parenchymal structures

**Figure 1:**
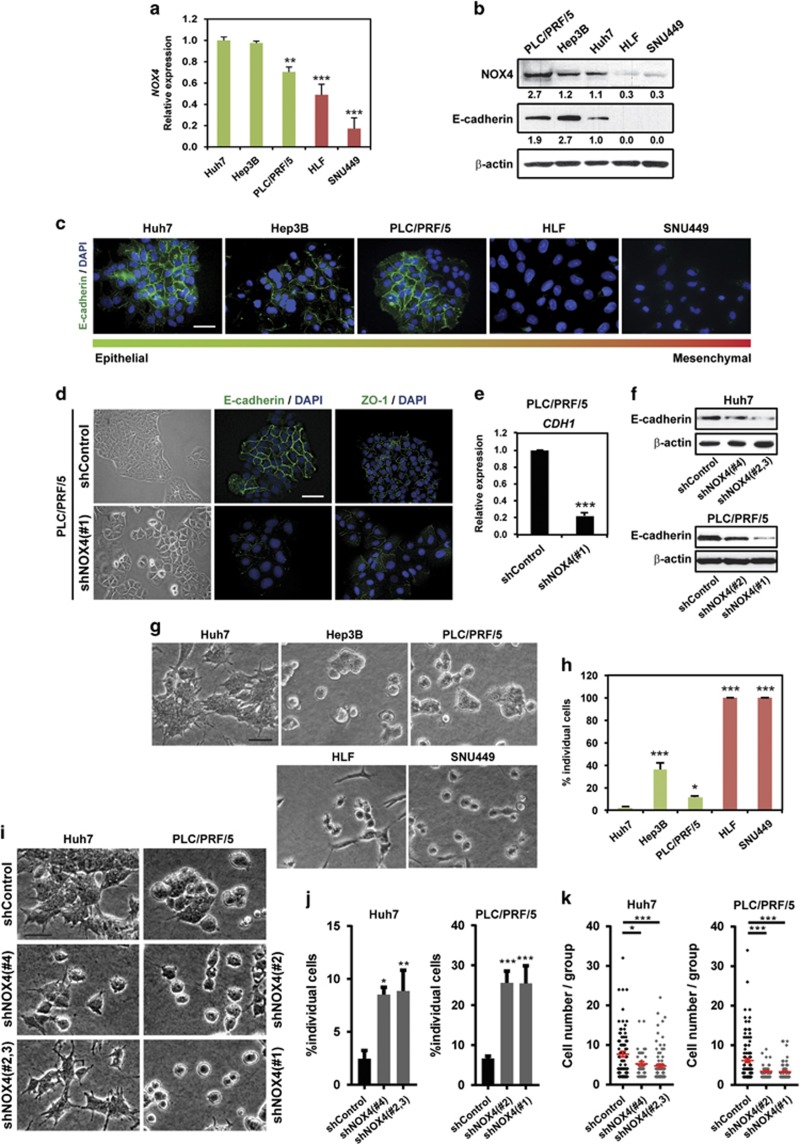
NOX4 regulates cell–cell adhesion in HCC cells. (**a**) *NOX4* expression levels by quantitative PCR. Data represent the mean±s.e.m. (*N*=4). (**b**) NOX4 and E-cadherin protein levels by western blot. β-actin was used as loading control. A representative experiment is shown (*N*=3). Quantification normalized by loading control is shown under each blot. (**c**) Immunofluorescence of E-cadherin (green) and DAPI (blue; scale bar, 50 μm). (**d**) Representative bright-field images of cells cultured at basal conditions on top of plastic (left). Cell–cell contact imaging by immunofluorescence of E-cadherin (green) and ZO-1 (green); DAPI (blue; *N*=3; scale bar, 50 μm; right). (**e**) Analysis of *CDH1* (E-cadherin) expression in PLC/PRF/5 cells by quantitative PCR. Data are mean±s.e.m. (*N*=4). (**f**) E-cadherin protein levels by western blot. β-actin was used as loading control. A representative experiment is shown (*N*=3). (**g**) Representative bright-field images of human HCC cells cultured on top of a bovine collagen I matrix (scale bar, 50 μm). (**h**) Quantification of the percentage of individual cells in each case. (**i**) Representative bright-field images of cells on top of a bovine collagen I matrix when NOX4 expression is silenced (scale bar, 50 μm). (**j**) Quantification of the percentage of individual cells in each case. (**k**) Number of cells per group. In (**h**, **j** and **k**) data are mean±s.e.m. (*N*=4). See also Supplementary Figures 1 and 2.

We have previously found that HCC patients express lower NOX4 protein levels when compared with healthy livers.^29^ Accordingly, analysis of NOX4 expression in different human HCC cell lines revealed important differences, from cells expressing high levels until cells that lack it (Figures 1a and b). As EMT is an important event in the acquisition of migratory abilities, we focused on EMT characteristics that could be associated with NOX4 expression. We analysed E-cadherin levels, both by immunofluorescence and western blot, and results showed that cells with high levels of NOX4 expression maintain epithelial features, whereas cells with low levels of NOX4 had lower levels of E-cadherin (Figures 1b and c). To analyse the effects of silencing NOX4 in those HCC cells that express high levels of NOX4, we performed stable transfections with specific NOX4 short hairpin RNAs (shRNAs) in PLC/PRF/5 and Huh7 cells (Supplementary Figure 1). Silencing of NOX4 correlated with decreased intracellular ROS production in all the cases (Supplementary Figure 2). PLC/PRF/5 displayed an epithelial phenotype forming parenchymal structures; however, when NOX4 was silenced the parenchymal phenotype was lost, correlating with decreased E-cadherin expression and lower cell-to-cell contact areas (Figures 1d–f). Decreased E-cadherin levels were similarly observed in NOX4 knocked down Huh7 cells (Figure 1f). When cells were cultured on a physiologically relevant pliable matrix, such as collagen I,^13, 30^ HCC cells that formed groups were those expressing higher levels of NOX4 (Figures 1g and h). Disruption of parenchymal structures was observed after NOX4 depletion (Figures 1i and j), resulting in groups containing fewer cells when compared with control (Figure 1k). These results show that NOX4 is necessary for maintaining an epithelial phenotype and a parenchymal structure in HCC cells.

### NOX4 is important for cell-to-matrix adhesion

**Figure 2:**
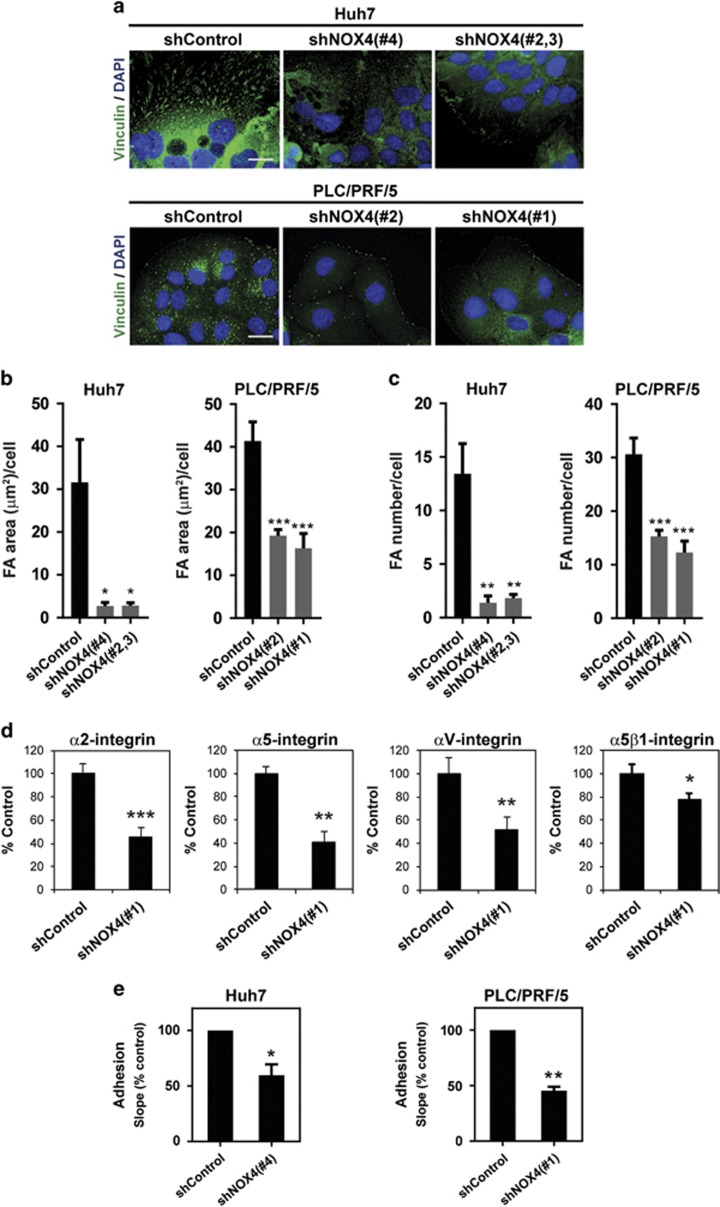
Loss of NOX4 disrupts cell–matrix adhesion in HCC cells. (**a**) Immunofluorescence of Vinculin (green) and DAPI (blue; *N*=3, 5 pictures/experiment; scale bar, 20 μm). (**b** and **c**) Vinculin image processing using ImageJ software: analysis of the area occupied by focal adhesions per cell (**b**), and quantification of the number of focal adhesions per cell (**c**). (**d**) Analysis of cell surface integrins using a α/β-Integrin-mediated cell adhesion array (Millipore). Selection of those integrins that presented significant changes in PLC/PRF/5 shNOX4(#1) cells (*N*=3). See the whole panel of integrins analysed in Supplementary Figure 4. (**f**) Real-time adhesion assay (xCELLigence system). Data are expressed as percentage versus control (unspecific shRNA-transfected) cells (*N*=3). In (**b**–**e**) data are mean±s.e.m. See also Supplementary Figure 3.

Next, we explored if NOX4 is also involved in cell-to-matrix adhesion. Analysis of Vinculin (Figure 2a) or phosphorylated Focal Adhesion Kinase (pFAK) (Supplementary Figure 3A) by immunofluorescence revealed lower levels of expression and a different distribution in NOX4-silenced cells, when compared with control cells. Both the area occupied by focal adhesions and the focal adhesion number per cell were significantly diminished in NOX4-silenced cells (Figures 2b and c; Supplementary Figures 3B and C). Using a α/β-integrin-mediated cell adhesion array, we confirmed decrease in levels of different integrin proteins on the cell surface. Significant lower levels of α2-, α5- and αV-integrins, as well as the complex α5β1-integrin, were observed when NOX4 expression was attenuated (Figure 2d; Supplementary Figure 4). These changes in focal adhesions and integrin levels were translated into a decrease in cell-to-matrix adhesion when NOX4 expression was lost, which was monitored in real-time using xCELLigence system (Acea Biosciences, San Diego, CA, USA) on cell culture plates (Figure 2e). These results suggest that NOX4 is also important for cell adhesion in HCC cells.

### NOX4 suppresses migratory/invasive potential

**Figure 3:**
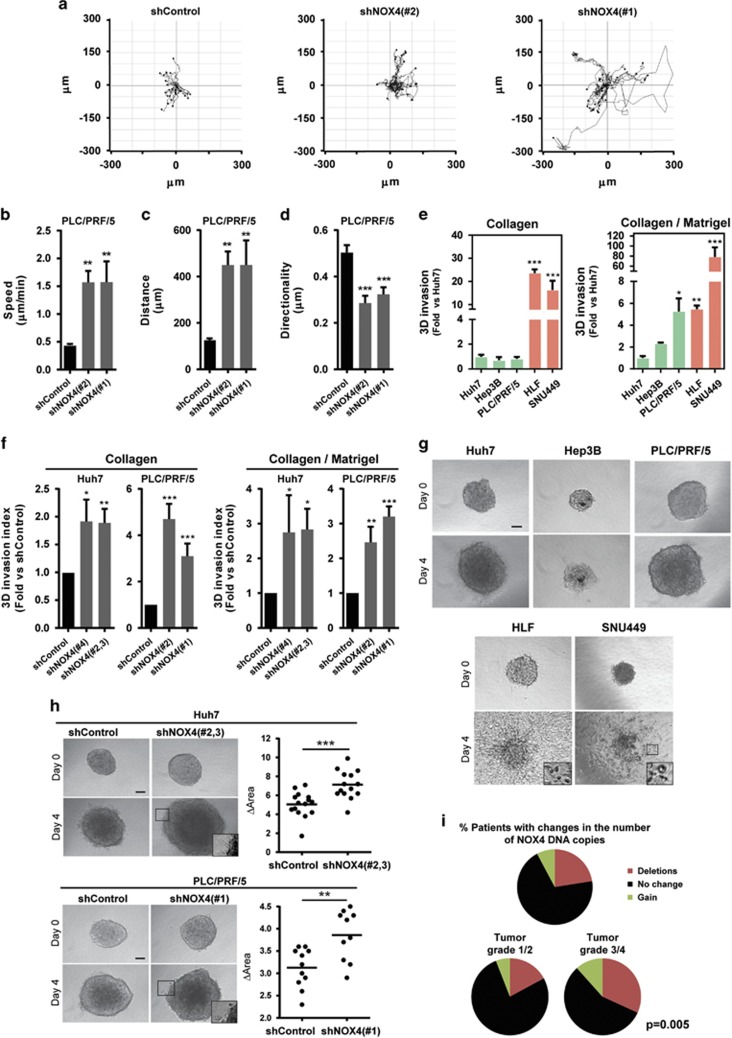
NOX4 suppresses the migratory/invasive potential of HCC cells. (**a**) Manual tracking of NOX4-silenced cells moving on a thick layer of collagen I/matrigel. Quantification of speed (**b**), distance (**c**) and directionality (**d**) of individual migrating cells. Data are expressed as mean±s.e.m. (20–30cells per condition). See also Supplementary Videos 1-3. Quantification of 3D invasion into a matrix of bovine collagen I (left) and collagen I/matrigel mix (1:1; right) of different human HCC cell lines (**e**) and of NOX4 knocked down Huh7 and PLC/PRF/5 cells (**f**). Data are expressed as fold increase versus Huh7 cells or shControl and are mean±s.e.m. (*N*=3–5). (**g**) Invasive growth analysed embedding cells as spheroids in a bovine collagen I matrix. Representative images at day 0 and 4 (scale bar, 100 μm). (**h**) Representative images and quantification of the increase in area of the spheroids from control (shControl) and NOX4-silenced (shNOX4) cells between day 0 and 4 after embedding them into collagen (scale bar, 100 μm). (**i**) Percentage of HCC patients showing *NOX4* deletion (23%) from the The Cancer Genome Atlas database (*N*=249). Proportion of HCC patients in tumour grade 1/2 (17% left) and tumour grade 3/4 (32% right) showing *NOX4* deletion.

We next analysed the migratory capacity of human HCC cells using wound-healing and chemotaxis assays. High levels of NOX4 were associated with lower migratory potential (Supplementary Figures 5A and B). Moreover, NOX4 silencing resulted in increased cell migration using both a wound-healing assay and real-time monitoring of cell migration either by xCELLigence system (Supplementary Figures 5C and D) or by time-lapse video microscopy analysis on a three-dimensional (3D) pliable matrix (Figures 3a–d; Supplementary Videos 1-3). To assess the invasive capacity of cells, we used an assay that measures individual cell invasion in three dimensions.^12, 13^ We used two different matrices, collagen I and collagen I/matrigel mixture to recapitulate matrix heterogeneity found in HCC lesions. Cells expressing low levels of NOX4 (HLF and SNU449) were the most invasive ones (Figure 3e). NOX4 loss increased the invasion potential of Huh7 and PLC/PRF/5 cells (Figure 3f). We further validated the relevance of NOX4 in HCC cell migration and invasion studying the behaviour of HCC cells cultured as spheroids embedded in collagen I. This assay measures proliferation and invasion over time (invasive growth).^31^ In a period of 4 days, cells with high levels of NOX4 showed lower invasive growth capacity (Figure 3g). Importantly, NOX4 silencing in Huh7 and PLC/PRF/5 cells increased their invasive growth capacity (Figure 3h). Furthermore, we found that 23% of HCC patients presented *NOX4* deletion, when *NOX4* DNA copy number alterations were analysed using The Cancer Genome Atlas database (http://cancergenome.nih.gov/) (*n*=249 patients). Interestingly, *NOX4* deletions were more frequently found in late stages of liver tumorigenesis (32% of patients in grade 3/4 versus 17% of patients in grade 1/2; Figure 3i).

We had previously reported a tumour-suppressive role for NOX4 via negative regulation of proliferation.^29^ With all these new data, we prove that NOX4 not only suppresses tumour growth, but also tumour invasion in HCC. Therefore, NOX4 may act as a tumour suppressor at different levels.

### NOX4 suppresses actomyosin contractility and amoeboid features

**Figure 4:**
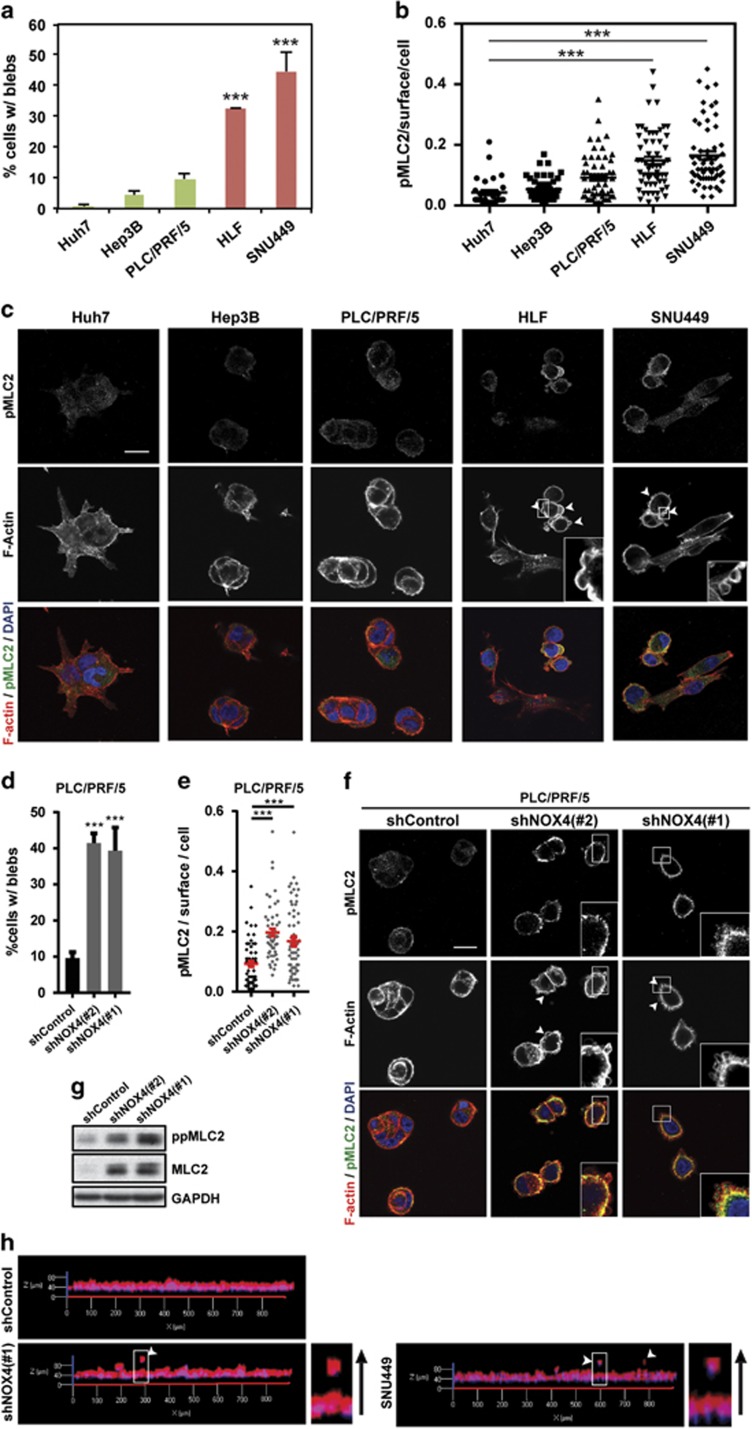
NOX4 suppresses actomyosin contractility in HCC cells. In all experiments cells were cultured on top of a bovine collagen I matrix. (**a** and **d**) Percentage of cells with blebs. (**b** and **e**) quantification of phosphorylated MLC2 (pMLC2) immunostaining intensity per cell surface. (**c** and **f**) Representative confocal images of pMLC2 (green), F-actin (red) and DAPI (blue) staining (*N*=4; scale bar, 20 μm, square with a higher magnification is shown for better visualisation). White arrows indicate blebs. Data in (**a**, **b**, **d** and **e**) are mean±s.e.m. (*N*=4, at least five pictures per experiment). (**g**) Phosphorylated and total MLC2 protein levels. GAPDH was used as loading control. A representative experiment is shown (*N*=3). (**h**) 3D imaging of PLC/PRF/5 (shControl and shNOX4) and SNU449 cells invading a collagen I/matrigel mix (1:1) matrix. Higher magnification is shown for better visualization. Black arrow indicates direction of invasion.

Actomyosin contractility is a major driver of invasion in 3D complex environments^32^ and higher levels of actomyosin contractility have been associated with lower levels of adhesion.^16, 17^ As actomyosin driven bleb-based migration has been shown recently to offer an advantage in many relevant physiological scenarios,^1, 14, 16, 17, 33, 34^ we first investigated if HCC cell lines can display amoeboid-blebbing behaviour, as this has not been described to date. We could observe bleb-like structures in some of the cell lines in our panel and this associated with increased levels of myosin light chain 2 (MLC2) phosphorylation. Note that we could not observe blebs when cells were part of parenchymal structures. More importantly, bleb formation and MLC2 phosphorylation levels were inversely associated with NOX4 expression (Figures 4a–c). In addition, NOX4 loss increased the percentage of cells with blebs and the levels of MLC2 phosphorylation (Figures 4d–g). Interestingly, after 3D imaging of NOX4-silenced invading cells, we could observe that they were rounded (Figure 4h). Similarly, amoeboid invasive behaviour was observed when imaging the most invasive cell line in our panel, SNU449 (Figure 4h). Therefore, these data show that NOX4, through its role as a negative regulator of actomyosin contractility, could regulate a contractile amoeboid phenotype and the invasive capacity of HCC cells.

### Overexpression of NOX4 suppresses actomyosin contractility and invasion

**Figure 5:**
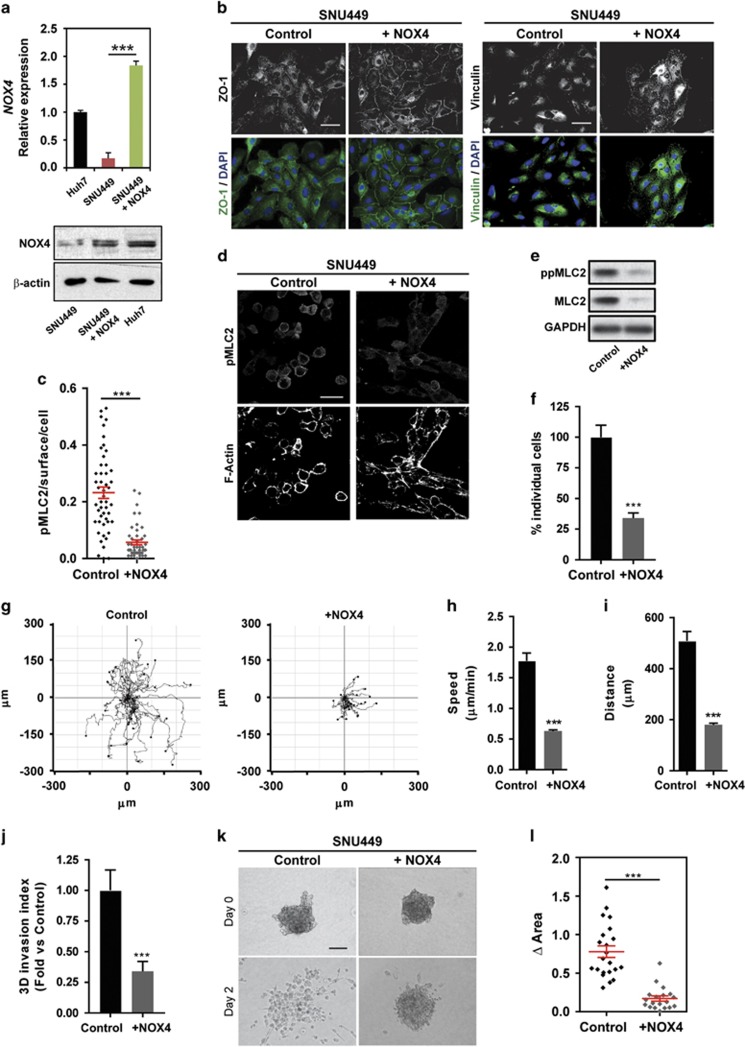
Overexpression of NOX4 in SNU449 cells switches to a parenchymal phenotype, and suppresses actomyosin contractility and invasive behaviour. (**a**) NOX4 mRNA (up) and protein (down) levels in SNU449 cells overexpressing NOX4 (+NOX4) and in Huh7 cells. Beta-actin used as a loading control. (**b**) ZO-1 (green) and Vinculin (green) immunofluorescence. DAPI (blue) to detect nuclei (*N*=3; scale bar, 50 μm). (**c**) Representative confocal images of immunostaining of pMLC2 and F-actin of HCC cells cultured on top of bovine collagen I matrix (*N*=3; scale bar, 50 μm). (**d**) Phosphorylated and total MLC2 protein levels. GAPDH was used as loading control. A representative experiment is shown (*N*=3). (**e**) Quantification of pMLC2 immunostaining intensity per cell surface. (**f**) Quantification of the percentage of individual cells cultured on top of a collagen I matrix. (**g**) Manual tracking of SNU449 cells moving on a thick layer of collagen I/matrigel. Quantification of speed (**h**) and distance (**i**) of individual migrating cells. Data are mean±s.e.m. (20–30cells per condition). (**j**) Quantification of 3D invasion into a bovine collagen I matrix. (**k**) Representative images of spheroids from control and NOX4 overexpressing cells at day 0 and 2 after embedding them into collagen I (scale bar, 100 μm). (**l**) Quantification of the increase in area of the spheroids between day 0 and 2. Data in (**a**, **c**, **f**, **j** and l) are mean±s.e.m. (*N*=3).

So far our data show that NOX4 plays a relevant role in maintaining epithelial and non-invasive features in HCC cells. Thus, we wondered whether re-expression of NOX4 could impact migratory and invasive properties. NOX4 overexpression in highly invasive SNU449 cells (reaching similar levels to those found in Huh7; Figure 5a) promoted the appearance of parenchymal structures, re-expression of important proteins for cell–cell interactions, such as ZO-1, and an increase in the number of focal adhesion complexes (Figure 5b). Furthermore, NOX4 overexpression induced a decrease in actomyosin contractility levels (Figures 5c–e), a decrease on the percentage of individual cells (Figure 5f), and reduced migratory (Figures 5g–i; Supplementary Videos 4 and 5) and invasive potential (Figure 5j). Moreover, cells cultured as spheroids into collagen I suffered a marked impairment on invasive growth when NOX4 was overexpressed (Figures 5k and l). All these data indicate that the sole overexpression of NOX4 is enough for maintaining parenchymal structures, increasing cell–substrate adhesion and suppressing actomyosin contractility and invasive behaviour in HCC cells.

### NOX4 regulates Rho GTPase expression

**Figure 6:**
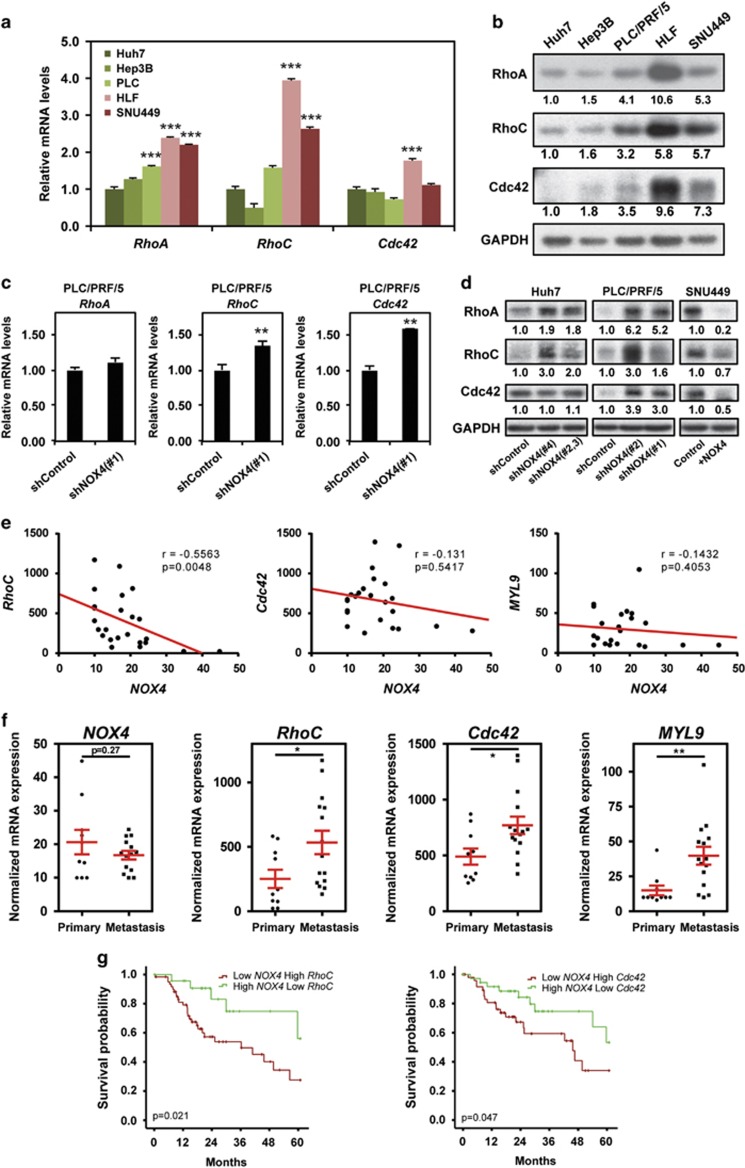
NOX4 suppresses the actomyosin regulators RhoA/C and Cdc42. (**a** and **c**) *RhoA*, *RhoC* and *Cdc42* GTPases mRNA expression levels analysed by quantitative PCR. Data represents the mean±s.e.m. (*N*=4). (**b** and **d**) Representative western blot of RhoA, RhoC and Cdc42 GTPases. GAPDH was used as loading controls (*N*=3–5). Quantification normalized by loading control is shown under each blot. (**e**) Scatter plots of *NOX4*, *RhoC*, *Cdc42* and *MYL9* (MLC2) expression correlation analysis using normalized mRNA expression data from GEO data sets (accession number GSE40367). (**f**) *NOX4, RhoC*, *Cdc42* and *MYL9* (MLC2) expression levels comparing primary tumour versus metastasis using same data set as in **e**. (**g**) Kaplan–Meier estimates of 5 years survival in The Cancer Genome Atlas HCC patients according to *NOX4* expression combined with *RhoC* and *Cdc42* expression.

Rho GTPases are crucial regulators of the cytoskeleton during cell migration and invasion.^32^ In order to understand how NOX4 is suppressing contractility and the invasive capacity of HCC cells, we analysed expression levels of different Rho GTPases that have been reported to be crucial for sustaining bleb-based migration and actomyosin contractility.^6, 7, 11^ We found higher messenger RNA (mRNA; Figure 6a) and protein levels (Figure 6b) of RhoA, RhoC and Cdc42 in cells with low NOX4 expression. In accordance, NOX4 loss in Huh7 and PLC/PRF/5 cells increased the levels of RhoA, RhoC and Cdc42 (Figures 6c and d) and conversely, overexpression of NOX4 in SNU449 increased their levels (Figure 6d). Furthermore, using publicly available data from gene expression analysis (Gene Expression Omnibus, GEO), we observed a significant inverse correlation between *NOX4* and Rho GTPases expression (*RhoC*, *Cdc42)*, as well as MLC2 (*MYL9*) expression (Figure 6e). Indeed, there is a significant increase in *RhoC*, *Cdc42* and *MYL9* expression levels in metastasis versus primary tumours (Figure 6f), indicating that drivers of actomyosin contractility are selected during HCC metastatic dissemination. Moreover, patients with low expression of NOX4 and high expression of either RhoC or Cdc42 had a significantly worse prognosis compared with patients that presented high expression of NOX4 and low expression of RhoC or Cdc42 (*n*=249 patients; Figure 6g). These results suggest that invasive and metastatic behaviour in HCC could be in part due to the regulation of Rho GTPase and MLC2 expression. These are all events suppressed by NOX4; therefore this is a mechanism by which loss of NOX4 could contribute to the progression of invasive HCCs.

## Discussion

**Figure 7:**
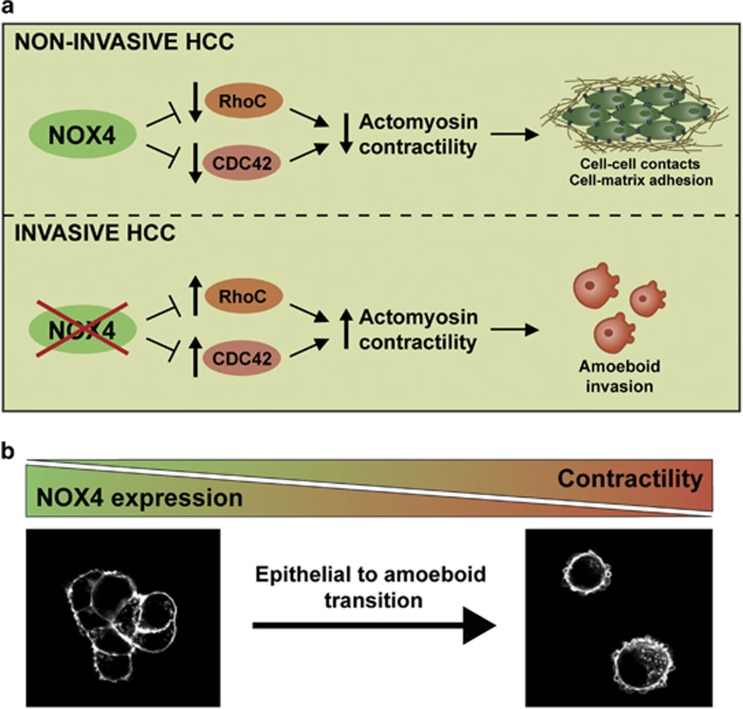
NOX4 suppresses amoeboid invasion in HCC. (**a**) NOX4 maintains epithelial parenchymal structures in non-invasive HCC. Loss of NOX4 promotes disruption of cell–cell and cell–substrate adhesion and increased RhoC and Cdc42 GTPases expression, favouring amoeboid invasion. (**b**) HCC aggressiveness is promoted through epithelial to amoeboid transition upon NOX4 loss.

Metastasis is a complex cascade of events, including abnormal migration and invasion of cancer cells. EMT has been described as a common step during metastatic dissemination of epithelial cancers.^21, 35^ Less is known about epithelial cancers transitioning towards amoeboid behaviour. Here we provide evidence that amoeboid invasion is, indeed, an efficient strategy used by HCC cells in physiologically relevant 3D environments. HCC is a poor prognosis tumour, presenting a high frequency of relapse and metastasis.^18^ In spite of recent advances in pharmacological treatments, HCC patient survival remains low.^36^ One major hallmark of an aggressive solitary HCC is its ability to migrate and invade, which increases the number of intrahepatic nodules (decreasing the transplantation possibilities), as well as its capacity to metastasize to other organs. Thus, a better knowledge about the mechanisms that regulate these processes may promote the development of effective approaches to reduce HCC mortality. Our work describes for the first time that NOX4 inhibits epithelial to amoeboid transition in HCC cells, maintains parenchymal structures, increases cell-to-matrix adhesion and inhibits actomyosin contractility through suppressing RhoC and Cdc42 GTPase expression (Figure 7a). Indeed, loss of NOX4 could contribute to HCC aggressive behaviour (Figure 7b).

The first NOX described was the respiratory burst NADPH oxidase complex, whose catalytic subunit is now known as NOX2. The other family members have been cloned and studied in the past 15 years.^37^ The functional analysis of the isoenzyme NOX4 revealed unique characteristics compared with other NADPH oxidases.^38^ NOX4 associates with their regulators (p22phox or Poldip2) in intracellular membranes, where the superoxide anion is rapidly converted to H_2_O_2_. Neither any of the known cytosolic oxidase proteins nor the GTPase Rac are required for its activity, which appears to be regulated mainly at the transcriptional level.^38, 39, 40^ Role of NOX4 in tumour progression is controversial and recent studies propose a role for NOX4 in supporting tumour migration.^41, 42^ However, although most of these studies were performed in two-dimensional migration systems, we have analysed the role of NOX4 in 3D systems that recapitulate interstitial tissue surrounding tumours, where we previously found that amoeboid migration is particularly efficient. Furthermore, it is worthy to note that NOX4 strongly mediates apoptosis and senescence in HCC cells,^43, 44^ as well as regulates homocysteine metabolism, which impacts on hepatic glutathione levels.^45^ Altogether suggest that NOX4 could have liver-specific functions, and its role may differ from other tumours, where it induces migration and cell survival.^41, 42, 46, 47^

Different data in the literature propose that NOX generated ROS, particularly those derived from NOX1, NOX2 and NOX4, are important regulators of the actin cytoskeleton and cytoskeleton-supported cell functions.^48^ Molecular mechanisms are not fully understood, although NOX4 may regulate, in a redox-dependent manner, integrins function.^49^ In vascular smooth muscle cells, NOX4 is required for focal adhesion formation and adhesion strength^50^ and for the activation of cytoskeleton regulators.^51^ However, one of the most relevant finding of our work is the role of NOX4 in regulating actomyosin contractility and Rho GTPases gene expression. Expression of NOX4 in different human HCC cell lines and in HCC patients inversely correlated with their levels of RhoA, RhoC and Cdc42 both at mRNA and protein levels. Furthermore, NOX4 knockdown increased RhoC and Cdc42 transcript levels, and RhoA, RhoC and Cdc42 protein levels. Many Rho GTPases seem to be regulated at their expression and not just by their activation status. Indeed, RhoA transcription is orchestrated by the Myc/Skp2/Miz1/p300 transcription complex.^52^ Interestingly, it has been described that upregulation of NOX4 in HCC cells is coincident with c-Myc downregulation,^43^ which would justify its capacity to inhibit Rho GTPases expression. The role of NOX4 on suppressing Rho GTPase expression and actomyosin contractility further reinforces its potential as a suppressor of liver tumour metastasis, as we show here that a significant increase in RhoC, Cdc42 and MLC2 expression levels is found in metastasis, when compared with primary liver tumours. Moreover, using publicly available data (The Cancer Genome Atlas) here we show that low expression of *NOX4* combined with high expression of either *RhoC* or *Cdc42* is associated with worse prognosis in HCC. Interestingly, it has been recently reported that low NOX4 expression is an independent predictor of both shorter relapse-free survival and shorter overall survival by using immunohistochemistry in tumour tissue from 227 HCC patients.^53^

ROS-mediated signalling has recently emerged as playing relevant suppressor actions, opposing to previous established ideas pointing ROS as mediators of tumour progression and antioxidants as anti-tumorigenic agents. Indeed, it has been described that antioxidants, such *N*-acetyl-cysteine or Trolox, increased the activation of RhoA and blocking RhoA signalling was enough to abolish antioxidant-induced cell migration and *in vivo* metastasis formation.^54, 55^ Furthermore, we have recently described how, in melanoma, RhoA activation and actomyosin levels are suppressed by ARHGAP5, a Rho GTPase-activating protein controlled by ROS.^13^ On the other hand, silencing of Nrf2, a master transcriptional activator of genes encoding enzymes that protect from oxidative stress, inhibits the ability of HCC cells to grow in soft agar and to form tumours.^56^ Activating mutations of Nrf2 occur at very early stages of the carcinogenic process, which suggests that the activation of Nrf2 pathway is mandatory for HCC progression.^56^

Our model fits with these last results, proposing that the expression of a gene involved in ROS-mediated signalling would be a suppressor factor in HCC. However, further analyses are needed to prove that the effects observed manipulating NOX4 expression are due to changes induced in ROS levels. Nevertheless, we show that NOX4 gene is deleted and/or expression downregulated in a significant percentage of HCC patients. Our data strongly supports that NOX4 suppresses liver tumour migration and invasion, which will need to be under consideration when using specific drugs to inhibit NOX4 in liver diseases. Finally, our work also adds HCC to the list of aggressive cancers that can engage into amoeboid dissemination strategies. Further work is needed to elucidate how many epithelial cancers can directly undergo epithelial to amoeboid transitions. Importantly, NOX4 could repress these processes in other epithelial tumours, and act as general metastasis suppressor.

## Materials and methods

### Cell models

Hep3B and PLC/PRF/5 cell lines were from the European Collection of Cell Cultures (ECACC, Porton Down, Salisbury, Wiltshire, UK). Huh7 and HLF cells (kindly provided by Dr Perales, University of Barcelona, Spain and Dr Giannelli, University of Bari, Italy, respectively) were from the Japanese Collection of Research Bioresources (JCRB Cell Bank, Ibaraki City, Osaka, Japan). SNU449 were from the American Tissue Culture Collection (ATCC, Manassas, VA, USA). Hep3B cells were grown in MEM medium supplemented with non-essential amino acids, SNU449 and HLF in RPMI medium, and Huh7, PLC/PRF/5 in DMEM medium, supplemented with 10% fetal bovine serum (FBS), in a humidified atmosphere at 37 °C, 5% CO_2_. Cell lines were never used in the laboratory for longer than 3 months after receipt or resuscitation.

### Cell culture on thick layers of collagen I

Fibrillar bovine collagen I (5005; PureCol, Advanced BioMatrix, San Diego, CA, USA) was prepared at 1.7 mg/ml in DMEM according to the manufacturer's protocol. After polymerisation (4 h–37 °C, 10%CO_2_) cells were seeded on top in medium containing 10% FBS and imaged after 24 h in culture.

### Knockdown assays

Huh7 and PLC/PRF/5 cell lines were stable silenced for NOX4 using four different shRNA plasmids (either alone or in combination) as described (see Supplementary Table I for shRNA sequences).^29^ Best-silenced clones were chosen and at least two different shRNA or combinations were used in each cell line.

### Overexpression assays

Lentiviral vector containing the human NOX4 sequence (NM_016931) was obtained from GE Healthcare (Little Chalfont, UK; OHS5898–219582330). Lentiviral supernatants were produced as previously described^57^ and used to infect SNU449 cells, which were then selected with 10 μg/ml blasticidin (from InvivoGen, Toulouse, France). The empty LentiORF-RFP-positive control (OHS5898) was used as a control.

### Analysis of gene expression

RNeasy Mini Kit (Qiagen, Valencia, CA, USA) was used for total RNA. Reverse transcription was carried out with random primers using High Capacity RNA to cDNA Master Mix Kit (Applied Biosystems, Foster City, CA, USA) following manufacturer's instructions. Expression levels were determined in a LightCycler 480 Real Time PCR System, using the LightCycler 480 SYBR Green I Master Mix (Roche Applied Science, Basel, Switzerland). See Supplementary Table II for primers sequences.

### Immunoblotting

Cells were lysed in RIPA lysis buffer 1 h at 4 °C. Western blotting was carried out as described previously.^29^ For Rho GTPases expression, cells were lysed in Laemmli sample buffer and sonicated 15 s before centrifugation.^13^ Antibodies used are summarized in Supplementary Table III.

### Immunofluorescence, confocal microscopy and image quantification

Epifluorescence microscopy studies were performed as described previously.^29^ ImageJ software (National Institutes of Health, Bethesda, MD, USA) was used to analyse the number, area and distribution of focal adhesions per cell from pFAK immunostaining TIFF images. DAPI staining was used to elucidate the number of cells.

For the immunostaining of cells seeded on top of a thick collagen I matrix, cells were fixed with paraformaldehyde and immunostained for rabbit anti-phospho-MLC2 (Ser19, Cell Signaling Technology, Danvers, MA, USA) and Alexa Fluor 546-phalloidin (Life Technologies Ltd, Paisley, UK) for F-actin detection as described.^13^ For imaging, collagen gels were transferred to glass-bottomed dishes and visualized on a Zeiss LSM 510 Meta confocal microscope (Carl Zeiss, Cambridge, UK) with C-Apochromat × 40/1.2 numerical aperture (water) and Zen software (Carl Zeiss). Confocal Z-slice images were analysed using ImageJ software. Phospho-MLC2 fluorescence signal was quantified calculating the pixel intensity in single cells relative to the cell area, determined using F-actin staining, using Fiji open source software (National Institutes of Health).

### Migration and invasion assays

Cell motility was examined by different methods: (i) transwell migration assay,^4^ (ii) wound-healing assay^58^ and (iii) real-time migration assay through the xCELLigence System (Acea Biosciences),^58^ and time-lapse microscopy and cell tracking of cells seeded on top of a thick layer of collagen I/matrigel.^13^ Spheroid cell culture was performed using the hanging drop method, as described previously by Del Duca *et al.*^59^ Once formed, spheroids were collected and resuspended in a collagen I solution (1.7 mg/ml in DMEM). 10% FBS-containing media was added on top (day 0) and phase contrast pictures were taken. For invasive growth quantification, increase on the area occupied by the spheroids between day 0 and 4 was calculated using ImageJ software. For 3D invasion assays,^13^ cells were resuspended in serum-free bovine collagen I solution at 2.3 mg/ml or in a solution of serum-free bovine collagen I/matrigel (Corning, New York, NY, USA) in a 1:1 proportion, to a final concentration of 14 000 cells per 100 μl of matrix and spun down, in a 96-well plate. After the matrix was polymerized, 10% FBS-containing media was added on top of the gel. The 3D invasion index was calculated as number of invading cells at 50 μm divided by the number of cells at the bottom. For 3D imaging of invading cells, cells were stained with Alexa Fluor 546-phalloidin (Life Technologies Ltd) and sequential *Z* sections were obtained using a Zeiss 710 confocal microscope. 3D reconstructions of invaded cells were made using Zen software.

### Adhesion assay

Real-time cell adhesion was examined using the xCELLigence System (Acea Biosciences). Overall 1.25 × 10^4^ cells per well were seeded onto an E-plate-VIEW 16 (Acea Biosciences), which features microelectronic sensors integrated on the bottom of the plate. Continuous values were represented as cell index, a dimensionless parameter reflecting a relative change in measured electrical impedance, and quantified as a slope (per hour) of the first 3 h.

### Integrin array

The α/β-Integrin-Mediated Cell Adhesion Array Combo Kit (ECM532, Merck Millipore, Billerica, MA, USA) was used according to the manufacturer's instructions. Each well containing mouse anti-alpha or anti-beta integrin received 100 μl containing 1.5 × 10^5^ cells, as did the bovine serum albumin-coated negative control wells. The plate was incubated for 2 h at 37 °C in 5% CO_2_ and washed with assay buffer. Cells were then stained with a Cell Stain Solution (provided in kit), incubated for 5 min and washed with dH_2_O. Extraction buffer (100 μl) was added to each well and left to shake for 5–10 min. Fluorescence was measured in Microplate Fluorescence Reader Fluostar optima (BMG Labtech GmbH, Ortenberg, Germany).

### Analysis of gene expression from human databases

From public database GEO (Gene Expression Omnibus, accession number GSE40367) we analysed expression of *NOX4*, *RhoC*, *Cdc42* and *MYL9* (MLC2) comparing HCC primary tumours and metastasis. Data were normalized using Gene Pattern (http://www.broadinstitute.org/cancer/software/genepattern/).

From The Cancer Genome Atlas database (http://cancergenome.nih.gov/), we extracted gene expression data of 249 HCC patients to analyse *NOX4*, *RhoC* and *Cdc42* expression in HCC outcome. Only patients whose tumour sample contained <15% of stroma were selected to avoid interference, as NOX4 is highly expressed in surrounding non tumoral tissue. We also extracted data concerning *NOX4* DNA copy number alterations from the same patients. Normalized mRNA expression data and *z*-scores for mRNA expression data and copy number alterations from GISTIC were downloaded from cBioportal^60, 61^ and analysed as described below.

### Statistical analyses

Two-sided Student's *t*-test versus control (unspecific shRNA-transfected) cells was used to generate *P*-values when only two groups were compared. Two-sided one-way analysis of variance with Dunnett's *post hoc* test versus Huh7 cells (when cell lines were compared) or versus shControl (when silencing shRNA were compared) was used to generate *P*-values (*P<0.05, **P<0.01 and ***P<0.001). Statistical analyses were performed using GraphPad Prism software (GraphPad, San Diego, California, USA). Data were represented as mean±s.e.m. In general, experiments were carried out at least three independent times with two to three technical replicates.

Pearson *χ*^2^ was used to determine *P*-values when comparing *NOX4* DNA copy number events and tumour grade.

For survival analysis, The Cancer Genome Atlas expression data were categorized using a *z*-score cutoff of 0. Kaplan–Meier method using the log-rank test was used to estimate survival curves. Pearson *χ*^2^ and survival analysis were performed using SPSS (IBM, North Harbour, Portsmouth, UK).
