# Supplementary material for: The NADPH oxidase NOX4 represses epithelial to amoeboid transition and efficient tumour dissemination
Source: Oncogene. 2016 Dec 12;36(21):3002–14. doi: 10.1038/onc.2016.454 (PMC5354266; doi:10.1038/onc.2016.454)
Supplement: Supplementary Figures [file onc2016454x1.docx]

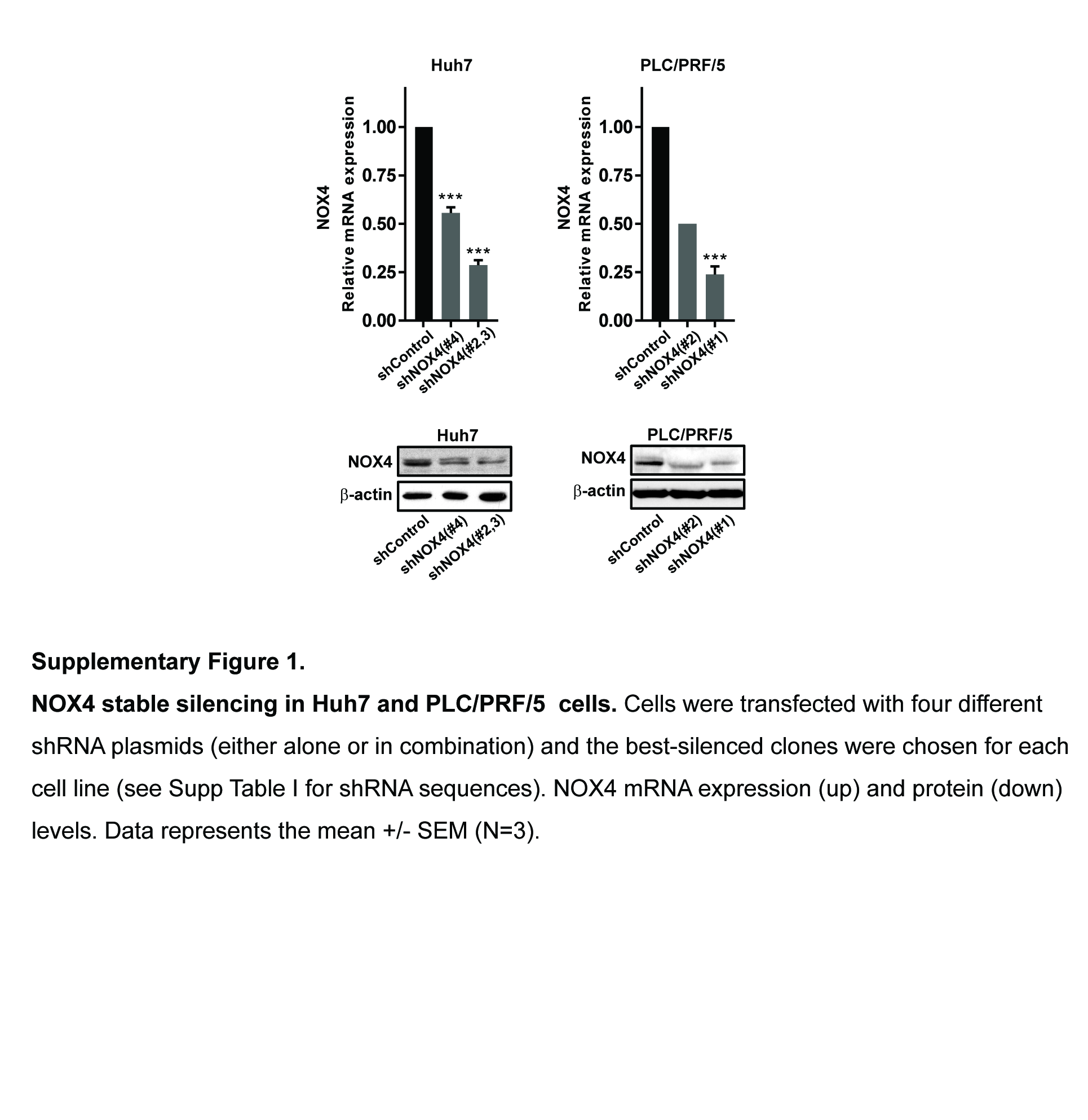


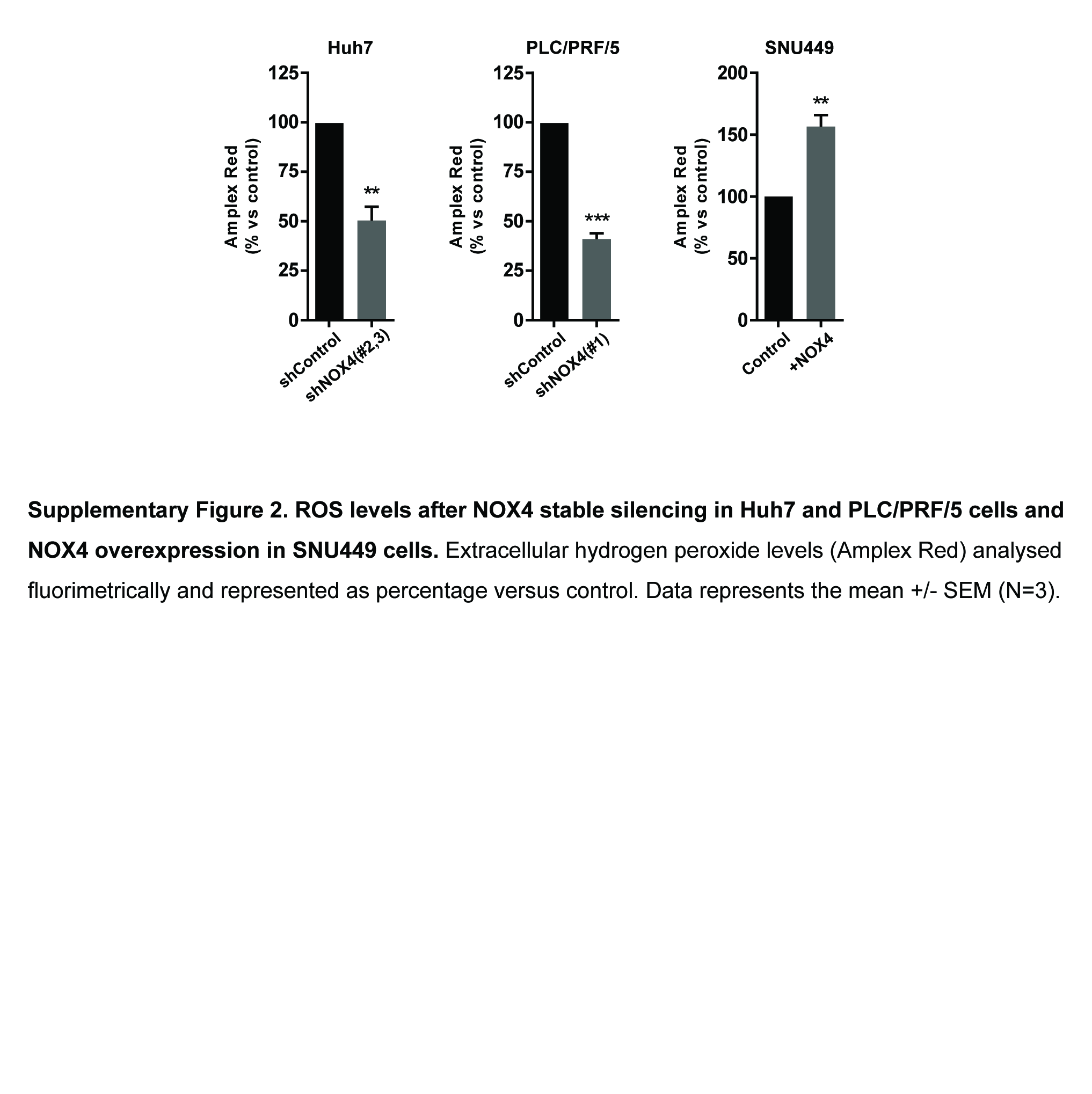


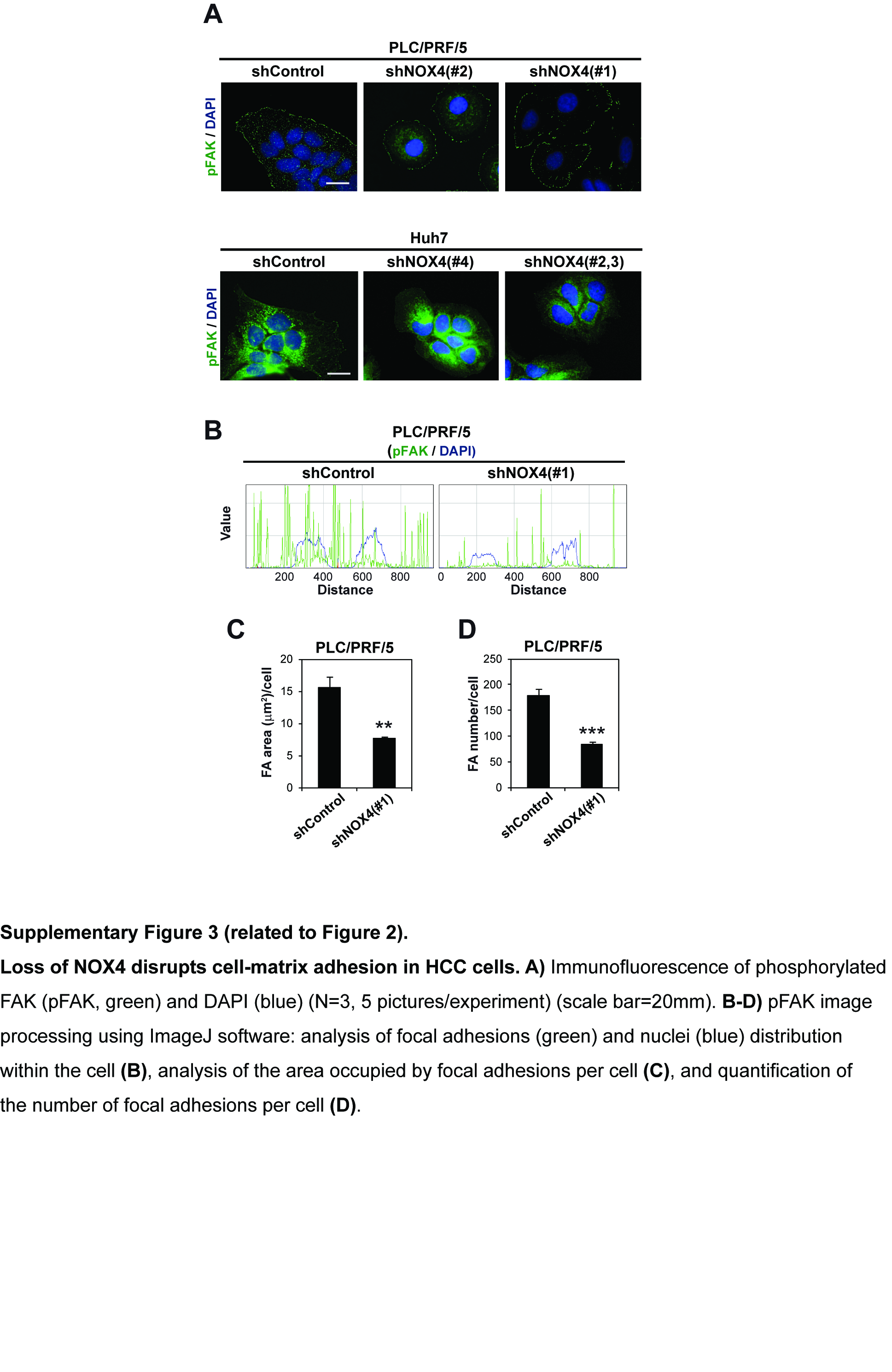


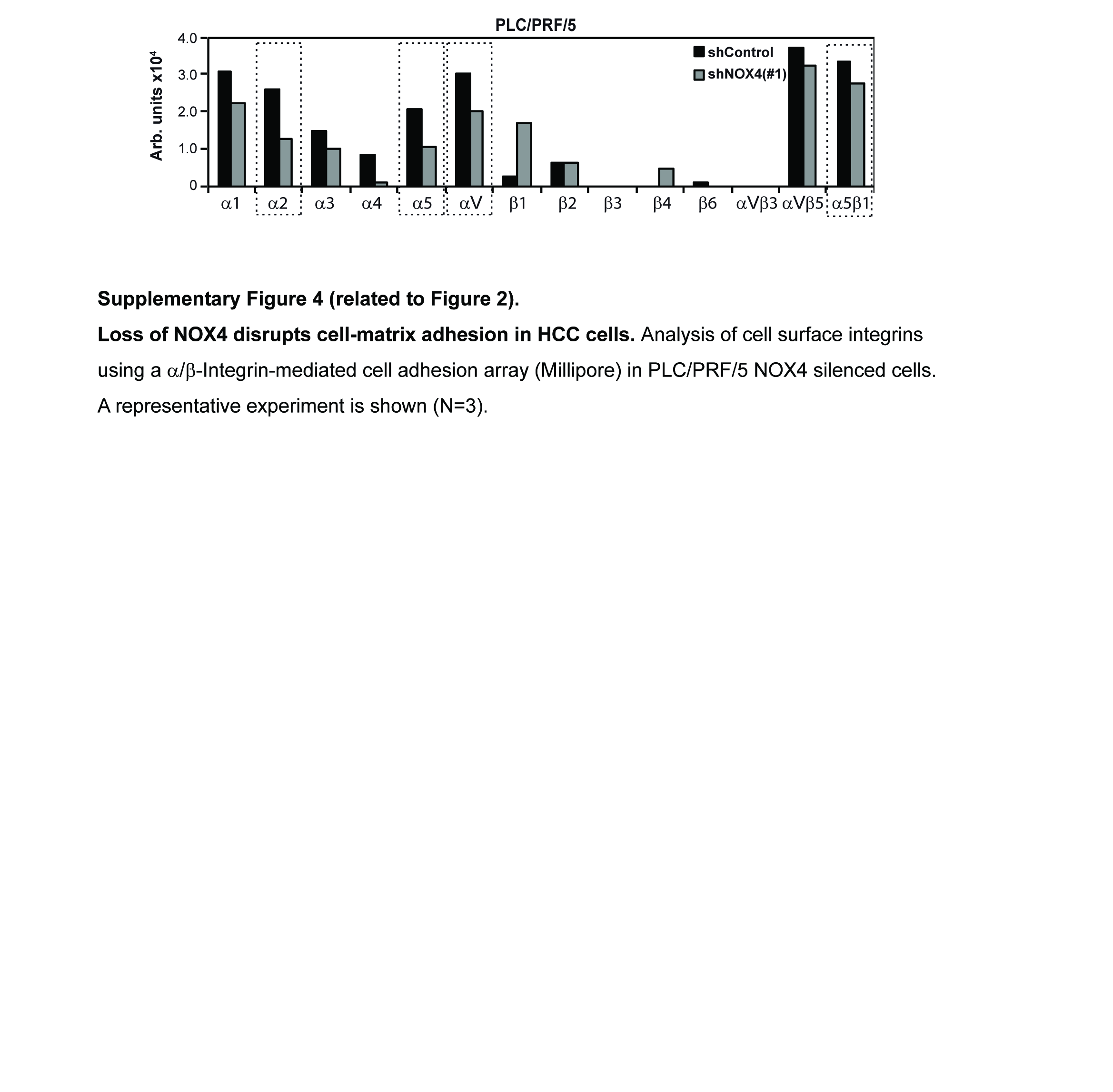


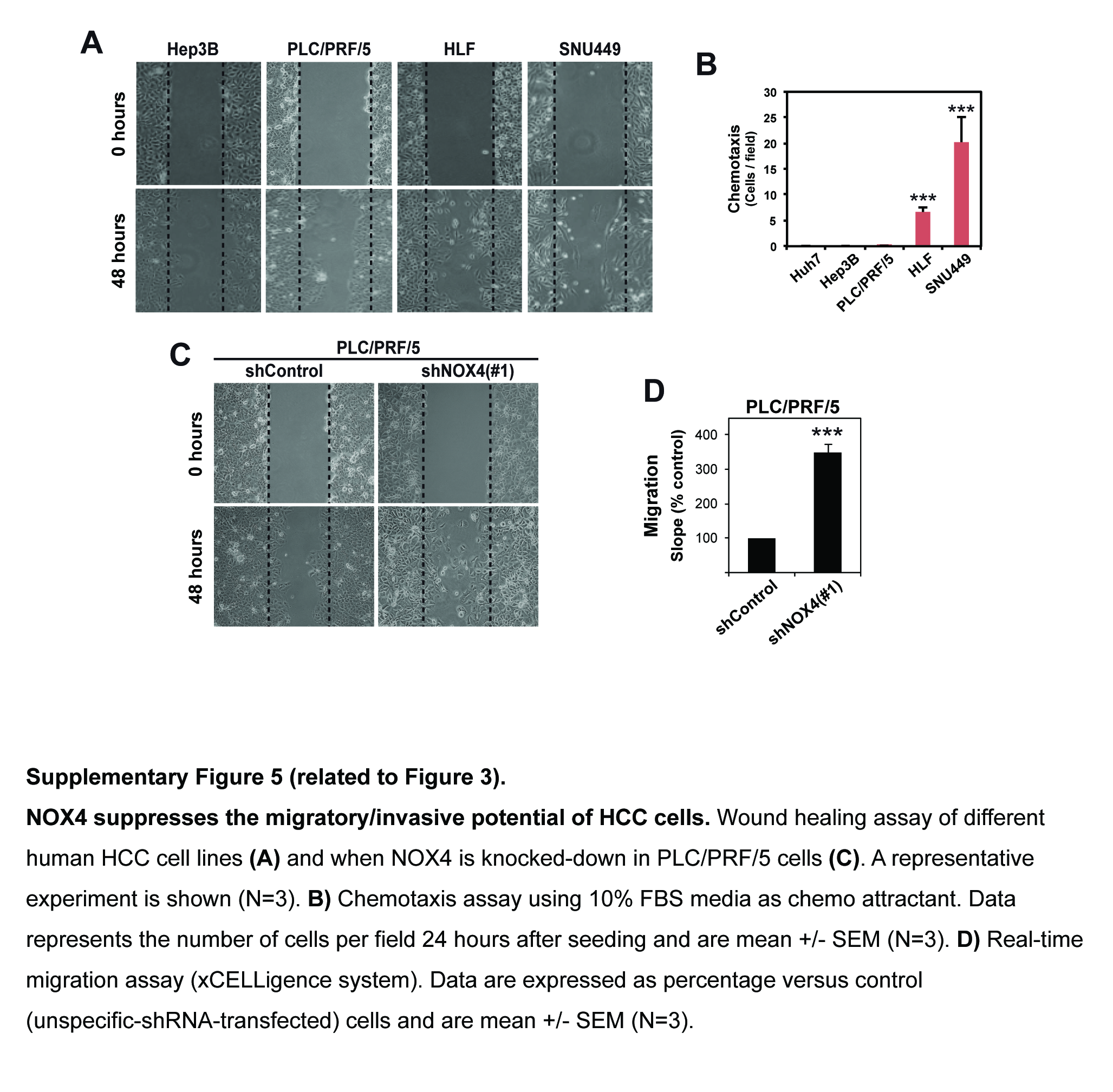


**Supplementary Movies**

**Supplementary Movie 1, 2 and 3, related to Figure 3.**

Time-lapse video microscopy with a 10x magnification objective of PLC/PRF/5 cells stably transfected with shRNA control (Movie 1) compared to shNOX4#2 (Movie 2) or shNOX4#1 (Movie 3) seeded on top of a thick layer of collagen I / Matrigel.

**Supplementary Movie 4 and 5, related to Figure 5.**

Time-lapse video microscopy with a 10x magnification objective of SNU449 cells control (Movie 4) compared to SNU449 cells overexpressing NOX4 (Movie 5) seeded on top of a thick layer of collagen I / Matrigel.
