# Supplementary material for: The NADPH oxidase NOX4 represses epithelial to amoeboid transition and efficient tumour dissemination
Source: Oncogene. 2016 Dec 12;36(21):3002–14. doi: 10.1038/onc.2016.454 (PMC5354266; doi:10.1038/onc.2016.454)
Supplement: Supplementary Tables [file onc2016454x2.docx]

| shRNA (gene) | Plasmid number | Sequence (5’-3’) |
| --- | --- | --- |
| Human NOX4 | #1 | CCGGGAGCCTCAGCATCTGTTCTTACTCGAGTAAGAACAGATGCTGAGGCTCTTTTTG |
|  | #2 | CCGGCCCTCAACTTCTCAGTGAATTCTCGAGAATTCACTGAGAAGTTGAGGGTTTTTG |
|  | #3 | CCGGCAGAGTTTACCCAGCACAAATCTCGAGATTTGTGCTGGGTAAACTCTGTTTTTG |
|  | #4 | CCGGGCTGTATATTGATGGTCCTTTCTCGAGAAAGGACCATCAATATACAGCTTTTTG |
| Supplementary Table I. shRNA sequences | | |

| **Gene (human)** | **Forward (5’-3’)** | **Reverse (5’-3’)** |
| --- | --- | --- |
| *RPL32* | AACGTCAAGGAGCTGGAAG | GGGTTGGTGACTCTGATGG |
| *CDC42* | CAGGGCAAGAGGATTATGACAG | GTTATCTCAGGCACCCACTT |
| *CDH1* | CCCAATACATCTCCCTTCACAG | CCACCTCTAAGGCCATCTTTG |
| *NOX4* | GCAGGAGAACCAGGAGATTG | CACTGAGAAGTTGAGGGCATT |
| *RHOA* | AGCTGGGCAGGAAGATTATG | CGTTGGGACAGAAATGCTTG |
| *RHOC* | CAAGACGAGCACACCAGG | AGCACTCAAGGTAGCCAAAG |

**Supplementary Table II.** Primers sequences used in LightCycler 480 SYBR Green System quantitative PCR

| Primary antibody | Purchased from |
| --- | --- |
| Mouse anti-β-actin | SIGMA |
| Rabbit anti-Cdc42 | Cell signaling |
| Mouse anti-E-cadherin | BD Pharmigen |
| Mouse anti-GAPDH | Merck Millipore |
| Rabbit anti-NOX4 | SIGMA-Genosys |
| Rabbit anti-phospho-FAK | Cell signaling |
| Rabbit anti-phospho-MLC2 | Cell signaling |
| Rabbit anti-phospho-Paxillin | Invitrogen |
| Rabbit anti-RhoA | Cell signaling |
| Rabbit anti-RhoC | Cell signaling |
| Mouse anti-Vinculin | SIGMA |
| Mouse anti-ZO1 | SIGMA |

**Supplementary Table III.** Antibodies used for immunoblotting and/or immunofluorescence
