# Supplementary material for: The NADPH oxidase NOX4 represses epithelial to amoeboid transition and efficient tumour dissemination
Source: Oncogene. 2016 Dec 12;36(21):3002–14. doi: 10.1038/onc.2016.454 (PMC5354266; doi:10.1038/onc.2016.454)
Supplement: Supplementary Information [file onc2016454x8.pdf]

STR Analyse AB140327
IDIBELL
Institute d'Investigacio Biomedica de Bellvitge, Spain

2014-05-07

STR-Data for: **HuH7**	date: 07.05.2014	data CLS/RIKEN/JCRB
**STR-Locus:**	**Wert 1 / Wert 2**	**Wert 1 / Wert 2**
Amelogenin	X,X	X,X
CSF1PO	11,12	11,12
D13S317	10,11	10
D16S539	10	10
D5S818	12	12
D7S820	11	11
THO1	7	7
TPOX	8,11	8,11
vWA	16,18	16,18
D3S1358	15	15
D21S11	30	30
D18S51	15	15
Penta E	11	11
Penta D	12	12
D8S1179	14	14,15
FGA	22,23	22,23

Data is 87,5 % identical with data from CLS. The cell line was authenticated as HuH7.
